# Can a Risk Factor Based Approach Safely Reduce Screening for Retinopathy of Prematurity?

**DOI:** 10.1155/2017/9372539

**Published:** 2017-01-09

**Authors:** K. M. Friddle, B. A. Yoder, M. E. Hartnett, E. Henry, R. J. DiGeronimo

**Affiliations:** ^1^College of Nursing, University of Utah, Salt Lake City, UT, USA; ^2^School of Medicine, Division of Neonatology, University of Utah, Salt Lake City, UT, USA; ^3^School of Medicine, Department of Ophthalmology, University of Utah, Salt Lake City, UT, USA; ^4^Intermountain Health Care, Institute for Health Care Delivery Research, Salt Lake City, UT, USA

## Abstract

*Objective.* Current American retinopathy of prematurity (ROP) screening guidelines is imprecise for infants ≥ 30 weeks with birth weights between 1500 and 2000 g. Our objective was to evaluate a risk factor based approach for screening premature infants at low risk for severe ROP.* Study Design.* We performed a 13-year review from Intermountain Health Care (IHC) data. All neonates born at ≤32 weeks were reviewed to determine ROP screening and/or development of severe ROP. Severe ROP was defined by stage ≥ 3 or need for laser therapy. Regression analysis was used to identify significant risk factors for severe ROP.* Results.* We identified 4607 neonates ≤ 32 weeks gestation. Following exclusion for death, with no retinal exam or incomplete data, 2791 (61%) were included in the study. Overall, severe ROP occurred in 260 (9.3%), but only 11/1601 ≥ 29 weeks (0.7%). All infants with severe ROP ≥ 29 weeks had at least 2 identified ROP risk factors. Implementation of this risk based screening strategy to the IHC population over the timeline of this study would have eliminated screening in 21% (343/1601) of the screened population.* Conclusions.* Limiting ROP screening for infants ≥ 29 and ≤ 32 weeks to only those with clinical risk factors could significantly reduce screening exams while identifying all infants with severe ROP.

## 1. Introduction

Retinopathy of prematurity (ROP) is the second leading cause of childhood blindness in the United States and the main cause of severe visual impairment associated with prematurity [1]. The National Eye Institute has estimated that, on an annual basis, 1100–1500 infants in the United States develop ROP severe enough to require medical treatment, of which 400–600 become legally blind [2]. The large majority of these infants are born at a birth weight less than 1250 g and a gestation < 29 weeks [3, 4].

Current screening criteria in the United States and many developed countries are based on gestational age (GA) and weight at delivery [5–8]. According to the most recent American Academy of Pediatrics (AAP) guidelines, screening examinations for ROP are recommended for all infants born at a GA ≤ 30 weeks or with a birth weight ≤ 1500 g, as well as for those > 30 weeks with birth weights between 1500 and 2000 g with an unstable clinical course [5]. It remains unclear what defines an “unstable clinical course,” making it difficult to determine which babies in this more mature subgroup should be screened. This uncertainty results in a number of babies undergoing retinal exams that are at minimal to no risk for severe ROP. Given that retinal examinations are costly and may be associated with pain and discomfort [9–12], efforts should continue to better define the target population of premature babies that would most benefit from ROP screening without missing at risk infants.

It has been our experience within the Intermountain Health Care (IHC) system that few babies born at greater than 29 weeks develop severe ROP (defined as stage 3 or higher or any stage requiring retinal laser surgery). More mature preterm babies, who do develop severe disease appear to have clinical risk factors that could be used to identify them for screening. The primary objective of this study was to describe the incidence of severe ROP in preterm infants less than or equal to 32 weeks GA born in the IHC system and from this population determine the incidence of severe ROP in those greater than or equal to 29 weeks. Additionally, we aimed to identify clinical risk factors in all infants with severe ROP to determine which, if any, could be used to better target screening examinations in lower risk, more mature preterm infants.

## 2. Methods

This study was a retrospective, Institutional Review Board approved review of data prospectively entered into electronic medical records from all neonatal intensive care units in the Intermountain Health Care (IHC) system. IHC, which owns multiple hospitals in the mountain west region, had a total of 4-level III and 1-level IV NICU all in Utah where ROP screening is routinely done during the study period. Deidentified data from 1 January 2000 through 30 December 2012 were reviewed on all premature neonates born at less than or equal to 32 weeks gestation to confirm retinal ROP screening as well as to identify the presence or absence of ROP. Given the imprecise nature of the AAP recommendations during the study period for screening larger babies at less risk for ROP between ≥29 and ≤32 weeks GA, at attending discretion not all infants within this select population were routinely screened. For those infants not screened during the initial hospitalization, an additional review was done through the electronic database for any subsequent diagnosis of ROP based on ICD9 codes or blindness. The program used for data collection is a web-based electronic medical record application that stores demographic and clinical information, such as history, physical examination results, laboratory data, problem lists, and discharge summaries. Data were managed and accessed by a single authorized data analyst (EH).

The international classification of ROP was used to document the zone and severity of disease and defines 5 stages of abnormal vessel development ranging from mild (stage 1) to retinal detachment (stage 5). Stage 3 is defined as abnormal intravitreal vasoproliferation and stage 3, is the stage in which in earlier days most treatment occurred [2, 13]. For this study, severe ROP was defined as stage 3 disease or greater or any ROP regardless of stage requiring laser retinal surgery (bevacizumab was not used for treatment during the study time period). In addition to demographic variables, the following data were extracted from the electronic medical record as possible indicators of an unstable premature infant course for identification of clinical ROP risk factors: a history of patent ductus arteriosus (PDA) treated surgically, necrotizing enterocolitis (NEC) and NEC surgery, duration of mechanical ventilation (MV), sepsis (confirmed by culture or a clinical course receiving a minimum of 7 days of treatment), use of darbepoetin/erythropoietin, and treatment with inhaled nitric oxide (iNO). Only surgical cases of proven NEC were used to reflect a more accurate diagnosis given the variability of coding for medical NEC in the database.

SPSS® 20 statistical software (IBM Corp. Released 2011. IBM SPSS Statistics for Windows, Version 20.0. Armonk, NY: IBM CORP) with a significance level set at <0.05 was used to analyze the data. Binary logistic regression analysis was done to determine variables significant in predicting the development of severe ROP. Unadjusted clinical variables were initially included in the regression model if they were significantly related to outcome and remained in the final model if they remained significant following adjustment for multiple variables (*P* < 0.05). The Hosmer-Lemeshow test was conducted to determine the model's goodness of fit. On the basis of the regression analysis a risk factor based model (ROP Risk Factor Score) was also developed to predict the likelihood of severe ROP or laser surgery. Based on derived odds ratios weighted scores for risk factors included gestational age in weeks (≤24 = 5, 25-26 = 3, 27-28 = 1, ≥29 = 0), birth weight in grams (<750 = 4, 750–999 = 2, >1000 = 0), MV > 5 days (3), NEC surgery (2), sepsis (2), PDA ligation (1), and inhaled nitric oxide (1). Receiver operating characteristic (ROC) curves and area under the curve (AUC) were developed using predicted probabilities derived from a generalized estimating equation model and from the cumulative ROP Risk Factors Score.

## 3. Results

**Figure 1:**
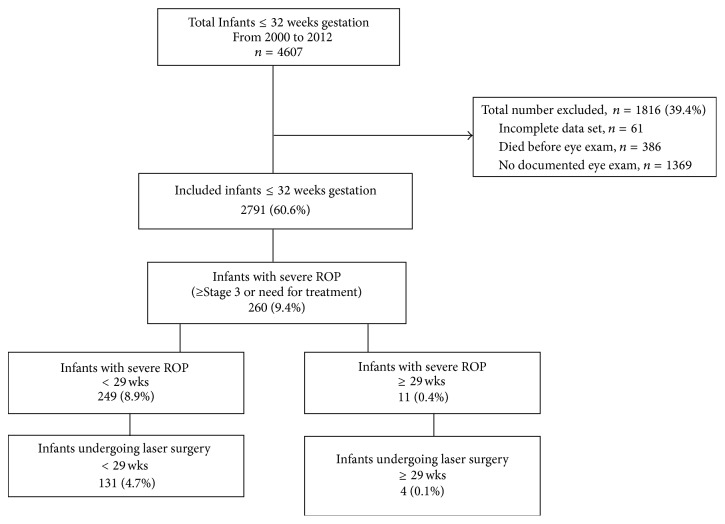
Study participants flow diagram. Infants were excluded due to lack of ROP screening data because of death, discharge, transfer, or ROP exam deemed unnecessary. Infants with severe ROP were categorized by gestational and/or birth weight group and then again by those in each group treated with laser surgery.

A total of 4607 premature infants born at 32 weeks GA or less were reported to the IHC database during the defined study period. The flow diagram of study participants is shown in Figure 1. Of the 2791 infants included in the final analysis, 260 (9.3%) had severe ROP. Out of the 1601 infants ≥ 29 weeks only 11 infants (0.7%) had severe ROP. Laser surgery was performed in a total of 135 (4.8%) babies with severe ROP, but only 4 (0.2%) were ≥ 29 weeks (Figure 1).

The study population had a median GA of 29 (IQR 27–31) weeks and birth weight of 1192 (IQR 900–1455) grams with slightly more males (53%) than females (47%). Babies that developed severe ROP were more immature (25 weeks; IQR 24–26) and smaller (704 grams; IQR 598–848) at birth and had a high risk of exposure to one or more defined risk factors.  Table 1 shows the clinical characteristics of the study population stratified by gestational age groups (<29 weeks and ≥29 weeks) and birth weights (<1250 g and ≥1250 g). Infants born at <29 weeks or <1250 g were significantly more likely to have severe ROP or undergo laser therapy and had significantly higher rates for sepsis, NEC surgery, PDA surgery, exposure to iNO, and darbepoetin/erythropoietin (data not shown) (Table 1). Additionally, duration of MV support was markedly longer. As noted in Table 1, only 6% of all screened infants ≥ 29 weeks had any noted ROP.

Table 2 lists the logistic regression coefficients for all babies with severe ROP or requiring laser therapy (Hosmer-Lemeshow test, *P* = 0.348). Early GA represented the highest risk factor for both outcomes followed by NEC requiring surgery and sepsis. MV also was predictive with a progressive increase in risk for each day of exposure. Inserting MV as a categorical variable (MV > 5 days) resulted in an OR for severe ROP of 5.6 (3.07–10.25, *P* ≤ 0.001) and for laser surgery 12.12 (3.59–40.9, *P* ≤ 0.001). Though significant in univariate analysis, darbepoetin or erythropoietin treatment was not independently significant by regression analysis. Additionally, BW was not used as a risk factor for our regression model due to its close association with GA.

The number of risk factors by gestational age for infants who developed severe ROP or required laser therapy is shown in Table 3. Two or more risk factors were present for 94% (245/260) of infants with severe ROP and 98% (132/135) of infants undergoing laser therapy. Only 1 of 260 infants with severe ROP (0.4%) had zero additional risk factors. That infant was 28 weeks and 1025 grams at birth and would have been screened based on those criteria alone.

Risk factors for all 11 infants > 29 weeks diagnosed with severe ROP, including the 4 that underwent laser surgery, are shown in Table 4. All but one of these infants had at least 2 of the identified ROP risk factors. The one infant with only a single clinical risk factor weighed 600 g at birth and would have been screened based on birthweight < 1250 g. Mechanical ventilation for >5 days was a risk factor in all 11 infants and sepsis was noted in 10 of the 11, whereas an operation for NEC or PDA was reported in only one infant. For screened infants ≥ 29 weeks, the rate for PDA surgery or NEC surgery was much lower than for screened infants < 29 weeks (Table 1).

We also analyzed risk factors and outcomes among the 1369 infants born between 29 and 32 weeks gestation not screened for ROP that survived and had a complete data set. Except for sepsis, ROP risk factors were extremely uncommon in this group (sepsis 67%, iNO 1%, NEC surgery 0.6%, PDA surgery 0.4%, days MV median 0). None of these infants were subsequently identified in the ICH data warehouse to have had ROP, laser surgery of the eye, or significant visual impairment.

**Figure 2:**
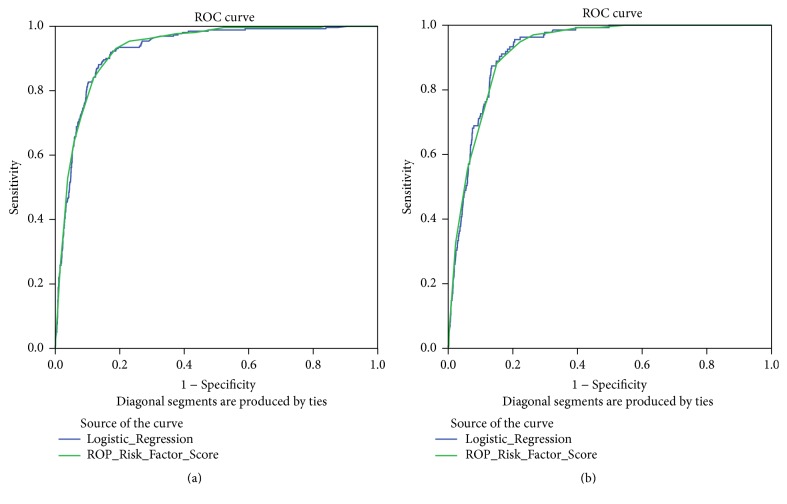
Receiver operating characteristic (ROC) curves showing sensitivity and specificity of predictive modeling for both diagnosis of severe ROP (a) and for treatment with laser therapy (b) using either the logistic regression model or the ROP Risk Factor Score.

Receiver operating characteristic (ROC) curves were generated from the logistic regression analysis and ROP Risk Factor Score as shown in Figure 2. Risk factors used in scoring included those described in Methods except for darbepoetin/erythropoietin. For both models area under the curve (AUC) values were quite high, exceeding 0.90, for prediction of severe ROP (Figure 2(a)) and laser therapy (Figure 2(b)). The near identical AUC values for the two models should be expected given that the ROP Risk Factor Score was weight-based derived from the regression model. Younger GA and MV > 5 days were the most heavily weighted factors in the ROP Risk Factor Score.

Utilizing our proposed risk based screening criteria, that is, screening all infants at a GA of < 29 weeks or a birth weight < 1250 g and selectively screening only those infants ≥ 29 and ≤ 32 weeks with the presence of at least one risk factor (sepsis, NEC or PDA surgery, ≥5days MV, or iNO) would have eliminated ROP screening in 21% (343/1601) of patients in our study population.

## 4. Discussion

In this 13-year study of all premature infants ≤ 32 weeks undergoing retinal screening exams and cared for in Intermountain Health Care neonatal intensive care units (*n* = 6), the incidence of severe ROP was 9.3% and the rate of retinal laser therapy was 4.8%. Every patient with severe ROP except one had at least one identified risk factor other than GA < 29 weeks or birth weight < 1250 g. This single infant qualified for screening based on a GA < 29 weeks. Among the 11 infants with severe ROP born at ≥29 weeks, all had a least 2 identified ROP clinical risk factors and therefore would have been screened based on our proposed screening strategy. Our findings suggest GA; BW combined with known risk factors may be an effective strategy for targeting ROP screening of premature infants born at ≥29 and ≤32 weeks and >1250 grams with an extremely low risk of missing severe disease.

Several recent publications have advocated for a risk factor based approach to ROP screening to limit unnecessary eye exams in low risk babies born at greater than 29 weeks gestation [6, 14–18]. Many, but not all, “developed” countries have secondary criteria for screening more mature babies, but suggested risk factors are not well defined [5, 7, 19]. van Sorge et al. reviewed all births prospectively enrolled in the Netherlands national ROP registry over a 12-month period for cases of severe ROP [16]. They found that if screening was limited to <30 weeks or <1250 g, as well as selected infants up to 32 weeks and 1500 g with one or more risk factors, no cases of severe ROP would be missed and screening could be reduced by 29%. The ROP risk factors used in their study included MV, sepsis, NEC, and postnatal corticosteroid or vasopressor medications. Yanovitch et al. reviewed the incidence and severity of ROP in a single US center over a 3-year period for infants with birth weights from 1250 to 1800 g [17]. From a total of 259 patients, they identified two patients with severe ROP. Both of these infants were under 1500 g and had at least 2 risk factors, which included sepsis, MV, prolonged antibiotics, multiple transfusions, and/or central line placement. Additionally, in an Australian regional review of 2292 babies > 30 weeks GA and 1250 g, no babies were found to have stage 3 or greater ROP and/or require laser surgery [14].

Several previous publications have noted that isolated cases of severe ROP may be missed in larger, more mature babies when using solely GA and birth weight screening criteria [15, 20, 21]. None of these reports, however, discussed ROP risk factors that may or may not have been present in their study populations and all are from 10 or more years ago when neonatal care practices may have differed. In a retrospective review of infants > 1250 g selectively referred for ROP screening, Hutchinson et al. found 7/1118 (0.6%) premature infants treated with laser therapy for severe ROP. No clinical details were described for any of these cases, nor the indications for ROP screening. Shah et al., in a single center retrospective review, reported 1/164 (0.6%) case of severe ROP in babies > 30 weeks GA. In the single case identified, neither birth weight nor specific clinical risk factors were discussed. Similarly, Lee et al. found a very low incidence of severe ROP outside of screening recommendations of <30 weeks or <1200 g in a review of infants from 14 Canadian neonatal units over a 2-year period, finding only one patient (1/2077). This one patient had a known complicated hospital course with MV and prolonged exposure to high inspired oxygen levels (FiO_2_) but more specific details of the hospital course were not given [21].

Based on the findings of our study and others [16–18], restricting routine screening to babies born under 29 weeks GA coupled with selective screening of babies up to 32 weeks with specific risk factors would significantly reduce ROP examinations when compared with current AAP and Canadian Pediatric Society recommendations. Implementation of this risk based screening strategy to the IHC population over the timeline of this study would have eliminated screening in 21% (343/1601) of those screened and potentially significantly more if applied to all babies born ≤ 32 weeks or 1250 g (given that 46% of the infants ≤ 32 were not screened). More restrictive ROP screening could potentially provide substantial health care savings as well as eliminate the pain and discomfort associated with unnecessary eye exams. Assuming that each infant in this relatively low risk group would have received an average of 3 screening exams, $543 per infant and $186,250 in total health care costs would be saved using this more restrictive screening approach (based on International Statistical Classification of diseases and related health Problems (ICD-9) codes of $213 for the initial and $165 for each follow-up ROP exam).

Limitations to this report include its retrospective nature and the fact that every neonate in our study population born at ≤32 weeks gestation was not screened for ROP, so it is possible that some infants ≥ 29 weeks without clinical risk factors could have developed severe ROP and were missed. Another limitation is that 67% of older babies ≥ 29 weeks whose records were reviewed did have sepsis identified as a risk factor but were not screened. Even though none of the infants identified with severe ROP in the screened cohort had only sepsis as an isolated risk factor, we cannot completely rule out the possibility that severe ROP could have been missed in some patients. Mild cases of ROP not requiring treatment could also have been missed as well as late cases that may have developed after a prolonged time period following NICU admission. In addition, the definition of ROP requiring treatment changed during the time period of analysis, as now infants with less than intravitreal vasoproliferation are included in treatment-eligible cases, which would have resulted in more babies being screened had this criteria been applied uniformly throughout the study period. We attempted to address these limitation by reviewing IHC warehouse data for all unscreened infants for any ICD 9 code associated with any stage of ROP or blindness, as well as for any retinal laser surgery. No infants were found to have any code for ROP, blindness, or laser surgery. Other study limitations include the lack of confirmation of either the LR probability equation or the ROP Clinical Risk Score for ROC/AUC analysis through analysis of a secondary data set. We currently are evaluating our risk factor based approach to screening for ROP in two additional population datasets outside of IHC in an effort to provide external validation to this clinical prediction model [22].

Future retrospective reviews by other centers using similar criteria will be important to publish prior to recommending any changes to current ROP screening guidelines and/or in designing prospective studies. Clearly, further validation of our experience within IHC needs to be done with careful monitoring for accuracy moving forward. Of interest, while all of the factors included in our Risk Factor Score significantly predicted severe ROP and/or need for laser surgery, several factors were commonly found including MV > 5 days that was present in all of the babies > 29 weeks. Sepsis was also common but, given its frequency as a risk factor, a better definition and tracking measure for infection likely needs to be developed. In general, though, it may be possible to create a more simplified risk factor based scoring system in future studies that would certainly help with its generalizability for use in future studies. Additional variables to include oxygen saturation limits based on GA and chronologic age will be important to consider in future studies looking to compare outcomes in different populations.

Recent evidence has shown the incidence of ROP may vary in countries based on the level of economic development, with larger more mature babies being at greater risk for severe ROP in less developed countries [6]. Whether identified clinical risk factors found in our study and others are also predictive of severe ROP in these countries remains unclear and needs validation. Additional research is also needed to evaluate other possible risk factors to optimize targeted ROP screening. Postnatal weight gain, hyperglycemia, and insulin-like growth factor levels have all been recently identified as potential predictors of severe ROP in at risk very premature infants [23–28].

Given the high rate of babies identified with sepsis in our study, having a better identification tool for this risk factor in future analysis may ultimately result in fewer lower risk infants ≥ 29 weeks needing to be screened. We chose to include all babies that received at least a 7-day course of antibiotics so as not to miss those identified with clinical sepsis regardless of whether or not they had a confirmatory positive blood culture. Also, while both PDA and NEC surgery appear to be important risk factors in high risk very preterm infants for the development of severe ROP, the fact that either surgery occurs much less frequently in lower risk older babies may make these less relevant risk factors in this subset of the population.

In summary, our results support the consideration of adjusting current ROP screening practices to a risk factor based approach for older premature infants (≥29 weeks) in the US and other developed countries. The implementation of such a strategy would significantly decrease unnecessary eye exams compared to current recommendations, potentially reducing physiological stress and pain to preterm infants as well as health care costs without missing cases of severe disease. We cannot say that this model is generalizable to all neonatal populations and consideration should be given toward developing or testing a strategy for each population based on resources available for perinatal and prenatal care.

## Figures and Tables

**Table 1:** Clinical characteristics of study population stratified by gestational age and birth weight.

	GA < 29 weeks (*n* = 1190)	GA ≥ 29 weeks (*n* = 1601)	*P*	BW < 1250 g (*n* = 1567)	BW ≥ 1250 g (*n* = 1224)	*P*	GA ≥ 29 weeks and BW ≥ 1250 g (*n* = 1125)
Any ROP *n*, (%)	648 (54)	100(6)^#^	<0.001	692 (44)	56(5)^#^	<0.001	40 (4)
Severe ROP *n*, (%)	249 (21)	11(0.7)^#^	<0.001	254 (16)	6(0.5)^#^	<0.001	3 (0.3)
Laser surgery *n*, (%)	131 (11)	4(0.2)^#^	<0.001	134 (9)	1(0.1)^#^	<0.001	1 (0.1)
Sepsis *n*, (%)	976 (82)	1068(67)^#^	<0.001	1250 (80)	794(65)^#^	<0.001	723 (64)
NEC surgery *n*, (%)	39 (3)	23(1)^#^	<0.001	45 (3)	17(1)^#^	<0.001	15 (1)
MV days (median, IQR)	19 (5–42)	1(0–4)^*∗*^	<0.001	11 (2–36)	1(0–3)^*∗*^	<0.001	1 (0–4)
iNO *n*, (%)	182 (15)	45(3)^#^	<0.001	183 (12)	44(4)^#^	<0.001	30 (3)
PDA surgery *n*, (%)	239 (20)	35(2)^#^	<0.001	250 (16)	24(2)^#^	<0.001	17 (2)

GA: gestational age; BW: birth weight; ROP: retinopathy of prematurity; NEC: necrotizing enterocolitis; iNO: inhaled nitric oxide; PDA: patent ductus arteriosus; IQR: interquartile range. Severe ROP was defined as ≥stage 3 ROP or any ROP requiring treatment. For all comparisons by both GA and BW, *P* < 0.001 (# is categorical variables Chi Squared Analysis and *∗* is MV days Mann–Whitney *U*, resp.).

**Table 2:** Combined logistic regression coefficients for infants with severe retinopathy of prematurity (ROP) or laser surgery.

	Severe ROP	*P* value	Laser surgery	*P* value
	(*N* = 260)		(*N* = 135)	
	Odds ratio (95% CI)		Odds ratio (95% CI)	
GA ≤ 24 weeks	49.4 (22.1–110.6)	<0.001	33.9 (18.7–364.6)	<0.001
GA 25 weeks	25.5 (11.6–56.1)	<0.001	25.0 (10.0–196.4)	<0.001
GA 26 weeks	22.5 (10.6–48.0)	<0.001	26.9 (11.1–206.7)	<0.001
GA 27 weeks	6.5 (3.0–14.9)	<0.001	11.7 (23.1–63.5)	0.01
GA 28 weeks	4.0 (1.7–9.4)	0.002	6.4 (1.6–39.8)	0.002
NEC surgery	2.9 (1.1–4.8)	0.038	8.8 (1.5–6.7)	0.003
Ventilator days^*∗*^	1.02^*∗*^ (1.01–1.03)	<0.001	1.01^*∗*^ (1.01–1.02)	0.001
Inhaled nitric oxide	1.6 (1.1–2.4)	0.014	1.6 (1.01–2.5)	0.046
Sepsis	1.8 (1.1–3.1)	0.019	7.0 (2.1–23.2)	0.001
PDA surgery	1.6 (1.1–2.3)	0.011	1.8 (1.1–2.7)	0.011

^*∗*^Increase in OR for each additional ventilator day. GA: gestational age; CI: confidence interval; NEC: necrotizing enterocolitis; PDA: patent ductus arteriosus.

**Table 3:** Gestational age related frequency of risk factors for severe retinopathy of prematurity (ROP) or laser treatment.

GA group (weeks)	Outcome	Number of risk factors				
		Zero	One	Two	Three	Four
≤24 (*n* = 160)	Severe ROP (*n* = 94)	0	3	33	42	16
	Laser (*n* = 53)	0	1	13	29	10
25 (*n* = 160)	Severe ROP (*n* = 58)	0	2	25	25	6
	Laser (*n* = 28)	0	1	11	12	4
26 (*n* = 208)	Severe ROP (*n* = 57)	0	2	25	23	7
	Laser (*n* = 31)	0	0	16	10	5
27 (*n* = 317)	Severe ROP (*n* = 27)	0	2	10	11	4
	Laser (*n* = 13)	0	0	3	7	3
28 (*n* = 345)	Severe ROP (*n* = 13)	1	4	2	3	3
	Laser (*n* = 6)	0	1	1	3	1
≥29 (*n* = 1601)	Severe ROP (*n* = 11)	0	1	5	3	2
	Laser (*n* = 4)	0	0	2	1	1
Total risk factors of screened Infants	Severe ROP	1	14	100	107	38
	Laser	0	3	46	62	24

**Table 4:** Characteristics of infants ≥ 29 weeks gestation who developed severe retinopathy of prematurity (ROP).

Year of birth	GA (weeks)	Birth weight (grams)	Worst stage ROP	Laser	Risk factors
2004	29	607	3	Yes	Sepsis, MV >5 days
2004	29	1240	2	Yes	Sepsis, MV > 5 days
2006	31	1235	3	Yes	NEC surgery, sepsis, MV > 5 days, iNO
2007	31	1845	3	Yes	Sepsis, MV > 5 days, iNO
2002	30	955	3	No	Sepsis, MV > 5 days
2002	31	600	3	No	MV > 5 days
2004	31	1332	3	No	Sepsis, MV > 5 days
2008	30	960	3	No	Sepsis, MV > 5 days, iNO
2009	29	1075	3	No	Sepsis, MV > 5 days, PDA ligation, iNO
2010	29	1320	3	No	Sepsis, MV > 5 days, iNO
2012	29	1040	3	No	Sepsis, MV > 5 days

GA: gestational age; MV: mechanical ventilation; iNO: inhaled nitric oxide; sepsis due to confirmed blood culture or a clinical course requiring ≥7 days of treatment; NEC: necrotizing enterocolitis; PDA: patent ductus arteriosus.
